# ID 357 - Fatores Associados aos Pareceres Técnicos Desfavoráveis dos Núcleos de Apoio Técnico do Poder Judiciário (NatJus): uma análise de 109.357 notas técnicas

**DOI:** 10.5327/2237-9622.2025.v34s1.357

**Published:** 2025-11-25

**Authors:** Mateus Bringel Oliveira Duarte, Marcelo Chioato, Francisco Diego Negrão Lopes, Roseane Eloiza Máximo Silva, Jonathas Torraca

**Keywords:** judicialização da saúde, avaliação da tecnologia biomédica, NatJus

## Abstract

**Introdução:**

Os Núcleos de Apoio Técnico do Poder Judiciário (NatJus) são unidades responsáveis por fornecer pareceres técnicos para auxiliar na decisão de processos judicializados relacionados à cobertura de tratamentos em saúde. O Conselho Nacional de Justiça tem atuado ativamente na padronização desses pareceres, os quais contemplam análises sobre aspectos técnicos e regulatórios das tecnologias avaliadas. Apesar da ampla utilização, a literatura ainda é limitada quanto à descrição do funcionamento dos NatJus e dos fatores que podem estar associados aos seus pareceres.

**Método:**

Realizamos um estudo transversal utilizando notas técnicas referentes a medicamentos, disponíveis no sistema e-NatJus, entre o período de 2018 a 2024 e abrangendo todo o território nacional. Após a coleta das notas técnicas, procedeu-se à extração semiautomatizada dos parâmetros analisados. Foram consideradas variáveis demográficas, clínicas (como a condição clínica e a urgência do caso), regulatórias (como o registro sanitário) e o conteúdo técnico da tecnologia avaliada (com base nos relatórios da Comissão Nacional de Incorporação de Tecnologias no Sistema Único de Saúde [Conitec] e nos protocolos do Ministério da Saúde [MS]). Os casos foram classificados de acordo com os capítulos do CID-10, correspondentes às condições descritas. Posteriormente, as notas foram analisadas conforme o parecer emitido, sendo classificadas como favoráveis ou desfavoráveis. Para avaliar os fatores associados aos pareceres desfavoráveis, utilizamos um modelo de regressão logística multivariado com imputação baseada em, para ajuste de variáveis mal definidas ou ausentes.

**Resultados:**

Foram analisadas 109.357 notas técnicas. A Região Sul apresentou o maior número de casos (40% do total). Entre as condições clínicas, 28% dos casos foram classificados com CID oncológico, sendo o grupo mais prevalente, seguido por doenças endócrinas e transtornos mentais (ambos com 11%). A maioria dos casos envolvia adultos (51%), enquanto 15% envolviam crianças e adolescentes. Não estavam previstos nos protocolos do Ministério da Saúde 76% dos casos, 15% foram classificados como de baixa evidência técnica e 28% como urgentes. Envolviam terapias recomendadas 19% dos casos, 22% não recomendadas e 58% não avaliadas pela Conitec, respectivamente.

Na análise dos fatores associados a pareceres desfavoráveis, observou-se que crianças (OR 0,52, P <0,001), regiões fora do Sudeste, condições dermatológicas (OR 0,12, P <0,001) e neoplásicas (OR 0,73, P <0,001) estiveram associadas a uma menor ocorrência de pareceres desfavoráveis. Por outro lado, a ausência de registro na Agência Nacional de Vigilância Sanitária – Anvisa (OR 1,12, P=0,009), tratamentos (OR 1,90, P <0,001), casos não previstos nos protocolos do MS (OR 1,87, P <0,001) ou no SUS (OR 1,87, P <0,001), não recomendados pela Conitec (OR 2,54, P <0,001), não avaliados pela Conitec (OR 1,31, P <0,001), sem evidência científica (segundo o parecerista, OR 2,54, P <0,001), e condições não urgentes (OR 6,88, P <0,001) foram associados a pareceres desfavoráveis.

**Conclusão:**

Nossos dados indicam que a evidência técnica, a urgência dos casos e as recomendações pela Conitec foram significativamente associadas à conclusão dos pareceres emitidos pelos NatJus.

